# A Call for Papers for the second special issue of SIGS from the Genomic Standards Consortium

**DOI:** 10.4056/sigs.1674461

**Published:** 2011-04-29

**Authors:** Dawn Field, Peter Sterk, Renzo Kottmann

**Affiliations:** 1 NERC Centre for Ecology and Hydrology, Maclean Building, Wallingford, Oxfordshire, United Kingdom; 2 Wellcome Trust Sanger Institute, Wellcome Trust Genome Campus, Hinxton, Cambridge, United Kingdom; 3 Microbial Genomics Group, Max Planck Institute for Marine Microbiology and Jacobs University Bremen, Bremen, Germany

Early issues of the Standards in Genomic Sciences (SIGS) journal have focused heavily on building a strong collection of genome notes compliant with the Minimum Information about a Genome Sequence (MIGS) checklist [1], in particular from the *Genomic Encyclopedia for Bacteria and Archaea project* (GEBA) [2]. This has resulted in SIGS quickly becoming the 3rd^th^ ranked journal of all time in terms of total number of newly published genome sequences. SIGS is now branching out to also include descriptions of metagenomes and pan-genomes.

As SIGS evolves it is now becoming a unique forum for publishing standards-compliant literature. Joint publication is a key step in the defining the shared interests and goals of communities. The November/December 2010 issue was a special issue containing meeting reports from five communities, including four from the Genomic Standards Consortium (GSC) [3].

In this issue, we present two types of articles new to SIGS that we believe will pave the way for similar submissions. Duhaime *et al.* review our current knowledge of a collection of marine phage genomes through the lens of MIGS-compliant fields, including habitat, geographic location, and host [4]. These authors contribute the first curation-based review paper to show that adherence to the GSC MIGS checklist enables a rich set of contextual data to be captured for marine phage. Likewise, SIGS now offers a forum for publishing multi-author, consensus-building papers that help define the work of the GSC and, hopefully in the future, related communities. Morrison *et al.* provide such a roadmap for the use of ontologies to mark up and interpret related data sources in new ways and introduce the Ontogrator software and website [5]. In a separate article, Castoe *et al*. present arguments from a consortium of zoologists advocating the sequencing of the garter-snake genome (*Thamnophis sirtalis*) [6].

## A call for multi-author, consensus-driven articles from the GSC and beyond

The longer-term goal of SIGS is to serve as an open-access, standards-supportive publication for all consensus-building communities working to develop standards and related infrastructure. To accelerate the expansion of the scope of SIGS, we are organizing a special issue coordinated by the GSC. To complement a range of submissions directly from the GSC, we welcome multi-author, consensus driven articles and 'roadmap' papers from communities working towards standardization of data within the genomic ('omic) sciences and related areas.

**Suggested topics include:**
- minimum information checklists
- ontologies
- file formats
- standards-compliant software and databases
- curation efforts to capture standards-compliant metadata
- Standard Operating Procedures (SOPS)
- data policies
- community-led efforts to undertake large-scale coordinated science projects (big science)

While reports describing new resources will be given priority, significant updates from more mature communities are ongoing and resources will also be considered. The deadline for submission to this special issue is May 30, 2011. Articles will be reviewed and published in the August/September issue of 2011.
